# Youth mental health care use during the COVID-19 pandemic in Alberta, Canada: an interrupted time series, population-based study

**DOI:** 10.1186/s13034-024-00785-1

**Published:** 2024-08-10

**Authors:** Matthew Joseph Russell, Liana Urichuk, Naomi Parker, Vincent Israel Opoku Agyapong, Katherine Rittenbach, Michele P. Dyson, Carla Hilario

**Affiliations:** 1 PolicyWise for Children & Families, 9925 109 St NW, Edmonton, AB T5K 2J8 Canada; 2 Department of Psychiatry, University of Alberta, 4-142A Katz Group Centre for Research, 11315 - 87 Ave NW, Edmonton, AB T6G 2H5 Canada; 3 Faculty of Social Work, University of Calgary, 2500 University Dr NW MacKimmie Tower (MT) 301, Calgary, AB T2N 1N4 Canada; 4 Faculty of Medicine, Dalhousie University, Abbie J Lane Building, 5909 Veterans Memorial Ln, Halifax, NS B3H 2E2 Canada; 5 Department of Psychiatry, Cumming School of Medicine, University of Calgary, 3330 Hospital Dr NW, Calgary, AB T2N 4N1 Canada; 6 Provincial Addiction and Mental Health, Alberta Health Services, 10th Floor, South Tower, 10030–107 Street NW, Edmonton, AB T5J 3E4 Canada; 7 School of Nursing, The University of British Columbia-Okanagan, ART360 (ARTS Building), 1147 Research Road, Kelowna, BC V1V 1V7 Canada

**Keywords:** Children, Youth, Mental health, Health care, COVID-19, Administrative data, Big data, Social determinants of health, Gender, Socioeconomic status

## Abstract

**Background:**

During the COVID-19 pandemic, youth had rising mental health needs and changes in service accessibility. Our study investigated changes in use of mental health care services for Canadian youth in Alberta before and during the COVID-19 pandemic. We also investigated how youth utilization patterns differed for subgroups based on social factors (i.e., age, gender, socioeconomic status, and geography) previously associated with health care access.

**Methods:**

We used cross-sectional population-based data from Alberta, Canada to understand youth (15–24 year) mental health care use from 2018/19 to 2021/22. We performed interrupted time series design, segmented regression modeling on type of mental health care use (i.e., general physician, psychiatrist, emergency room, and hospitalization) and diagnosis-related use. We also investigated the characteristics of youth who utilized mental health care services and stratified diagnosis-related use patterns by youth subgroups.

**Results:**

The proportion of youth using mental health care significantly increased from 15.6% in 2018/19 to 18.8% in 2021/22. Mental health care use showed an immediate drop in April 2020 when the COVID-19 pandemic was declared and public health protections were instituted, followed by a steady rise during the next 2 years. An increase was significant for general physician and psychiatrist visits. Most individual diagnoses included in this study showed significant increasing trends during the pandemic (i.e., anxiety, adjustment, ADHD, schizophrenia, and self-harm), with substance use showing an overall decrease. Mortality rates greatly increased for youth being seen for mental health reasons from 71 per 100,000 youth in 2018/19 to 163 per 100,000 in 2021/22. In addition, there were clear shifts over time in the characteristics of youth using mental health care services. Specifically, there was increased utilization for women/girls compared to men/boys and for youth from wealthier neighborhoods. Increases over time in the utilization of services for self-harm were limited to younger youth (15–16 year).

**Conclusions:**

The study provides evidence of shifts in mental health care use during the COVID-19 pandemic. Findings can be used to plan for ongoing mental health needs of youth, future pandemic responses, and other public health emergencies.

**Supplementary Information:**

The online version contains supplementary material available at 10.1186/s13034-024-00785-1.

## Introduction

Use of services for mental health challenges has grown among young people for decades [1]. In the first wave of the COVID-19 pandemic these challenges deepened. Canadian youth (aged 15–24) reported poorer mental health compared to their pre-pandemic mental health status [2], with about 70% of young people with and without pre-existing mental health conditions reporting deterioration in mental health [3]. The COVID-19 pandemic also exacerbated existing issues with supports and resources [4]. Public health measures during the pandemic changed health systems and services, disrupting the ways in which youth were able to access mental health care. For example, health services were shut down or significantly limited at the start of the pandemic, simultaneously shifting towards online, telehealth care [5]. The disruption and altered states of social and health service delivery can be especially harmful for youth with existing mental health conditions and complex service needs who access mental health services more often [6, 7].

Research to date suggests a significant rise in mental health disorders during the COVID-19 pandemic, with documented increases in depression, anxiety, and adjustment disorders (e.g., post-traumatic stress disorder) among young people [8–12]. Growing evidence also suggests that youth had increased Attention-Deficit/Hyperactivity Disorder (ADHD) symptoms due to the shift to online learning [13]. Evidence is less clear on substance use. Some studies found that youth who reported poorer mental health during the pandemic were also more likely to report increased substance use [14], while other research suggests that substance use declined overall among youth [10]. Another concern related to rising mental health needs is that they are linked to increased mortality [15, 16]. As such, growing mental health needs experienced by youth during the pandemic may have led to corresponding increases in mortality.

Some youth faced additional, layered challenges due to social factors that have been linked to mental health care use and access, including social support, income, and gender [17, 18]. They draw attention to how social and economic factors shape young people’s health [19, 20]. For example, some studies suggest that young women had greater symptoms of stress, anxiety, and depression in the pandemic than young men [21]. Increased social burdens placed on women during the pandemic and increased help-seeking behaviors for women have been used to explain these differences [22–24]. Income may have affected youth as well because unemployment and food insecurity have been linked to stress in children, and both increased in the multiple waves of the pandemic [25]. As such, we might expect increases in mental health needs among youth living in conditions of poverty [26]. On the other hand, wealthier Canadian families might have accessed services more because they had greater access to mental health care [27, 28].

This study investigates which groups of youth used mental health services before and during the pandemic and how they accessed the care.

### Research objectives

This research addresses knowledge gaps in the mental health service use of Canadian youth (15–24 years old) in Alberta during the COVID-19 pandemic. The objectives of the study are to investigate: (1) patterns of health care service use by youth for mental health reasons before and during the COVID-19 pandemic, (2) changes in youth mortality outcomes during the pandemic, and (3) if specific youth subpopulations (e.g., young women and men) had different patterns of use.

We expected an immediate decrease in mental health care use at the beginning of the pandemic, followed by an increase in mental health care use during the pandemic. Other investigations explored mental health care use trajectories in Alberta during the pandemic.

## Methods

### Overview of design

This is a population-based, cross-sectional study of health care administrative data documenting mental health service use in Canada before and during the COVID-19 pandemic. The study investigates social and economic factors that shape young people’s mental health [20], including socioeconomic status, age, and gender.

### Datasets

This study focused on provincial health care administrative data due to variability in health care service delivery across Canada. We derived variables and comparison rates from health care administrative data from the Government of Alberta’s Ministry of Health (i.e., Ambulatory Care, Inpatient [DAD], Longitudinal Demographic Profile [LDP], Population Registry, Practitioner Claims, and Vital Statistics—Deaths datasets) [29], publicly reported deaths from the Government of Alberta [30], and population and Census data from Statistics Canada [31–33]. Alberta health care data were provided as part of the Health Research Data Access pathway that provides data access to academic researchers in Alberta [29].

### Primary and secondary mental health diagnoses

Youth were classified by the presence of a mental health diagnosis during any of their psychiatrist, general physician (including pediatricians), emergency room, or hospitalization health care visits. Diagnoses in this study were identified by the research team based on known prevalence and clinical significance. The five primary diagnoses included: mood, anxiety, ADHD, substance use, and adjustment disorders. The two secondary diagnoses were: schizophrenia and self-harm. We note that self-harm is a behavior stemming from a variety of mental health related diagnoses. Diagnoses were derived based on previous case definitions (supplement Table S1 for code list) [34–38]. To represent the main reason youth were seen, only the most responsible diagnosis field was included. The exception is self-harm, which is only included as a later diagnosis in Alberta. We report how many diagnoses were given for each type of health care visit in supplement Table S2. We also report what type of health care visit was seen for diagnoses in supplement Figs. S1, S2, S3, S4, S5, S6, S7. We focus on general physician and psychiatrist visits as the primary type of visit for all diagnoses except self-harm.

### Study population

The study population consists of youth in Alberta, Canada who were: (1) between 15 and 24 years of age in the fiscal years 2018/19 to 2021/22 (April 1st to March 31st); (2) registered in the Alberta Population Registry; and (3) had a health care visit for a primary mental health diagnosis. The youth population that met these criteria resulted in a sample that ranged from 81,830 to 100,681 youth per year (see Table 1). Health care registration ensured residence in Alberta and allowed comparison of rates.

**Table 1:** Characteristics of Alberta youth using mental health care for the studied diagnoses

Youth characteristics	2018/19	2019/20	2020/21	2021/22
Alberta Youth 15–24 years old N	526,097	530,851	532,705	535,968
% with mental health diagnosis	15.55	16.08	16.94	18.78
Confidence Interval (95%)	(15.46–15.65)	(15.98–16.18)	(16.84–17.05)	(18.68–18.89)
N	81,830	85,358	90,266	100,681
Mean Age (15–24 years old)	19.74	19.71	19.69	19.60
Confidence interval (95%)	(19.72–19.76)	(19.69–19.73)	(19.67–19.71)	(19.58–19.62)
Standard deviation	2.84	2.86	2.86	2.87
Women/girls (vs. Men/boys) %	58.50	58.74	60.97	62.03
Confidence interval (95%)	(58.16–58.84)	(58.41–59.07)	(60.65–61.29)	(61.73–62.33)
N	47,871	50,142	55,035	62,453
Rural (vs. urban) %	17.76	17.43	16.96	16.57
Confidence interval (95%)	(17.5–18.02)	(17.17–17.68)	(16.71–17.2)	(16.34–16.8)
N	14,534	14,876	15,306	16,683
Lowest SES %	25.32	24.56	23.09	22.36
Confidence interval (95%)	(25.02–25.63)	(24.27–24.86)	(22.81–23.37)	(22.1–22.62)
N	20,181	20,443	20,343	21,965
Middle lower SES %	20.65	20.26	19.89	19.34
Confidence interval (95%)	(20.37–20.93)	(19.99–20.53)	(19.62–20.15)	(19.1–19.59)
N	16,456	16,861	17,520	19,001
Middle SES %	16.63	17.10	17.56	17.63
Confidence interval (95%)	(16.37–16.89)	(16.84–17.35)	(17.31–17.81)	(17.4–17.87)
N	13,251	14,229	15,468	17,322
Middle upper SES %	17.68	17.89	18.39	18.96
Confidence interval (95%)	(17.42–17.95)	(17.63–18.15)	(18.14–18.65)	(18.72–19.21)
N	14,091	14,893	16,205	18,626
Highest SES %	19.72	20.19	21.07	21.70
Confidence interval (95%)	(19.45–20)	(19.92–20.46)	(20.8–21.34)	(21.44–21.96)
N	15,718	16,805	18,562	21,312
Missing SES N	2133	2127	2168	2455
Income support Use %	11.27	11.42	9.00	8.55
Confidence interval (95%)	(11.05–11.49)	(11.21–11.63)	(8.82–9.19)	(8.37–8.72)
N	9222	9749	8127	8605

Youth are 15–24 years old in each year. SES = Neighborhood socioeconomic status. SES is the average income quintile for the youth’s neighborhood reported in the 2021 Census. The youth population in Alberta (top) is the denominator for youth with mental health diagnosis. The percent of youth with mental health diagnoses is the denominator for other youth characteristics

### Youth characteristics

Youth characteristics were chosen based on data availability and previously observed relationships with health care access [19]. Individuals with missing information were not included in related analyses.

1. *Age* is the rounded years of age as of the preceding fiscal year end (March 31).
2. *Gender* is the most frequently reported value (*men/boys or women/girls*). Although this variable has been captured as biological sex, we define it as gender in this study due to its interaction with socially constructed behaviours (i.e., the research focuses on mental health care use, which is known to be linked with gender-related social factors) [21, 39].
3. *Neighborhood socioeconomic status* is the average income quintile for the youth’s neighborhood (*lowest, middle lower, middle, middle upper, or highest*) reported in the 2021 Census [33]. Quintiles represent the proportion of youth in each Alberta dissemination area with the average income. The income may represent youth, family, or parental income.
4. *Rural (vs. urban)* is whether a youth lived in a Dissemination Area (a small geographic area set by Statistics Canada) that is not a Census Metropolitan Area or Agglomeration (larger population centres) in the study period [40].
5. *Income support use* includes youth who were ever recorded as using Government of Alberta income supports in the study period.

### Outcome: health care use

Outcomes are based on the use of Alberta health care through general physician (including pediatrician) visits, psychiatrist visits, emergency room visits, or hospitalizations. Mental health care use is defined as having at least one visit in any of the four health care types with a primary or secondary mental health diagnosis. We focus on most responsible (main) diagnoses in this paper and include other diagnoses in the supplement. We split mental health care use by type (e.g., emergency room or hospitalization) in Sect. ‘‘Health care use’’.

### Analysis

SAS Enterprise Guide 8.3 was used for analyses. We defined the *pre-pandemic* period as 2018/19 to 2019/20 and *during-pandemic* as 2020/21 to 2021/22. First, descriptive statistics show the characteristics of youth with mental health care use in each year (Table 1). Ninety-five percentile confidence intervals are provided.

Next, we report the monthly rate per 100,000 Alberta youth using mental health care by psychiatrist visits, general physician visits, emergency room visits, or hospitalizations. The rate is based on the estimated youth population, as a linear trend over the year. We used an interrupted time series design, using segmented regression to test for changes (crude rate per 100,000 youth) from pre- to during-pandemic. This design has often been used to test for changes in health care use during the pandemic [41–44]. We included a term for the trend before the pandemic, the month the pandemic started, the presence of the pandemic, and the trend over the pandemic. We tested for autocorrelation with the Durbin-Watson statistic and corrected when necessary.

Our next analyses calculated rates per 100,000 youth-years and standardized based on Statistics Canada youth population numbers. First, we calculated the monthly rate of youth with mental health care diagnoses. For each diagnosis, we used an interrupted time series design, using segmented regression with the same design as above. We provide quarterly most-responsible diagnoses and other diagnoses in the supplement. Second, we investigated yearly all-cause mortality rates for youth with any primary or secondary mental health diagnosis and compared them to deaths for other youth. We derived other youth death rates by subtracting deaths seen in our cohort from publicly reported youth deaths. To understand which youth were most impacted, we investigated yearly all-cause (any reason) mortality among Alberta youth by each diagnosis, using the rate per 100,000 youth with a diagnosis in the year. Finally, to examine subpopulations’ use of mental health care, we stratified diagnoses based on youth subgroups to investigate yearly rates of Alberta youth mental health care use.

## Results

### Characteristics of youth using mental health care

The Alberta youth population increased from 526,097 to 535,968 over the study period (Table 1). The proportion of youth using mental health care significantly increased from 15.6% to 18.8%, with the largest, significant increase during the pandemic (from 16.9% in 2020/21 to 18.8% in 2021/22). Over time, youth that used mental health care were on average younger; more likely to be girls/women; less likely to be from rural settings; more likely to be living in a higher socioeconomic status neighborhood; and less likely to be on income support. The clearest shifts were to a higher proportion of girls/women than boys/men and less use of mental health care for poorer youth. We also note that telehealth visits increased greatly during the pandemic [45]. Unfortunately, we are unable to quantify this increase in this analysis.

### Health care use

**Fig. 1:**
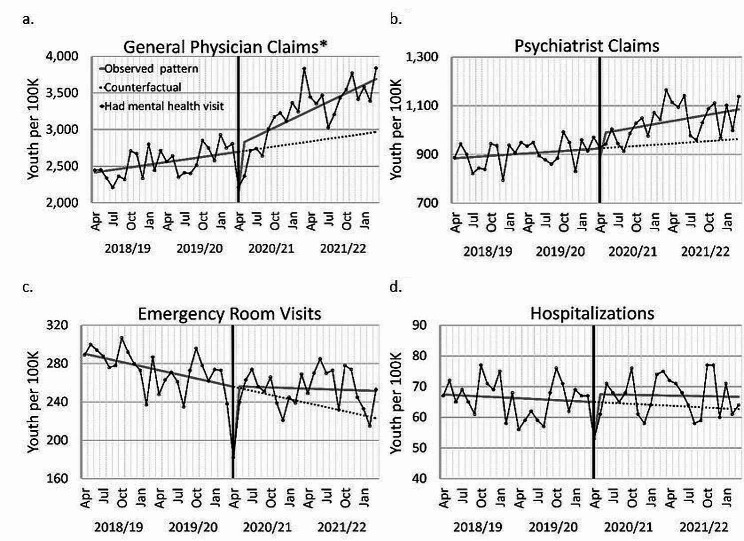
Monthly rates of Alberta youth using mental health care services by type of visit. Youth are 15–24 years old in each year. Visits are for studied mental health care diagnoses. The black line with points is the actual rate. Points are monthly, but labels only shown quarterly. Solid grey lines are regression lines for the observed patterns. The vertical black line marks the start of the COVID-19 pandemic. The dashed line is the continued trend from the pre-pandemic pattern (the counterfactual). *General physician claims include general and family physicians, and pediatricians

The most to least accessed type of mental health care was general physicians, psychiatrists, emergency room, and hospitalization (Fig. 1). The largest changes in mental health care use during the COVID-19 pandemic were in general physician use, with a drop at the start of the pandemic (− 657.35 per 100,000 youth; p < 0.001) followed by a trend increase across the remainder of the pandemic (+ 27.24 per month; p < 0.001). Psychiatrist mental health care use also increased across the pandemic (+ 58.04, p < 0.01), following a drop at the start of the pandemic (− 106.1, p < 0.005). Emergency room use and hospitalizations declined at the start of the pandemic (− 74.52; p < 0.001; − 14.53; p < 0.05; respectively). A significant increasing trend before the pandemic was observed for general physician use, with a significant decreasing trend for emergency room use. All other patterns were not significant. See supplement Tables S3-S4 for numbers and detailed model parameters.

### Diagnoses

**Fig. 2:**
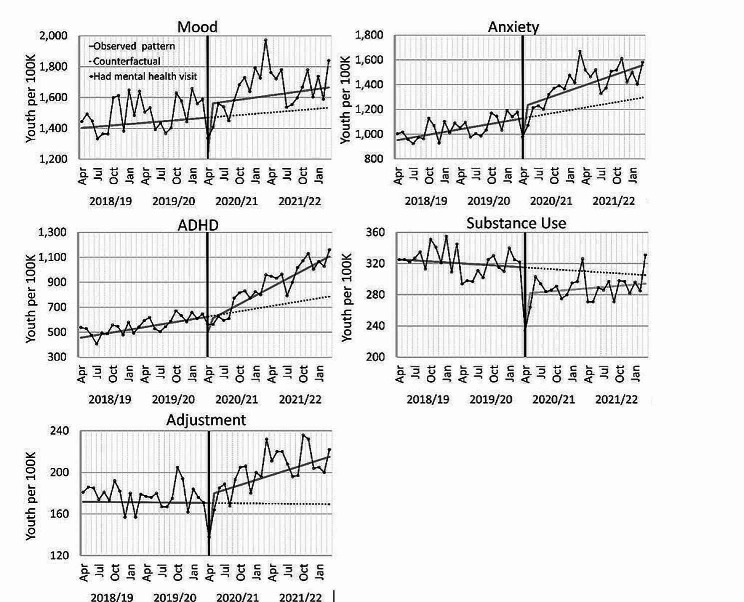
Monthly rates of Alberta youth using mental health care services for primary diagnoses of study. The black line with points is the actual rate. Points are monthly, but labels only shown quarterly. Solid grey lines are regression lines for the observed patterns. The vertical black line marks the start of the COVID-19 pandemic. The dashed line is the continued trend from the pre-pandemic pattern (the counterfactual)

For the primary diagnoses of study (Fig. 2), health care use for mood disorders showed a drop at the start of the pandemic (− 321.53 per 100,000 youth; p < 0.001), with a significant average increase across the pandemic period studied (+ 95.79; p < 0.05). Service use for anxiety disorders had a drop at the start of the pandemic (− 256.59; p < 0.01), with a near significant trend increase across the pandemic (+ 7. 45 per month; p = 0.052). Service use for ADHD had a drop at the start of the pandemic (− 91.89; p < 0.05), with a trend increase across the pandemic (+ 15.8 per month; p < 0.0001), but a delayed start prior to the increase (− 46.08; p < 0.05). Substance use had a drop at the start of the pandemic (− 48.51; p < 0.01) and an overall decrease in the pandemic (− 34.65; p < 0.001). Adjustment disorder had a drop at the start of the pandemic (− 45.89; p < 0.001), with a trend increase across the pandemic (+ 1.87 per month; p < 0.001). ADHD and anxiety disorders showed significant signs of growing before the pandemic. All other patterns were not significant.

**Fig. 3:**
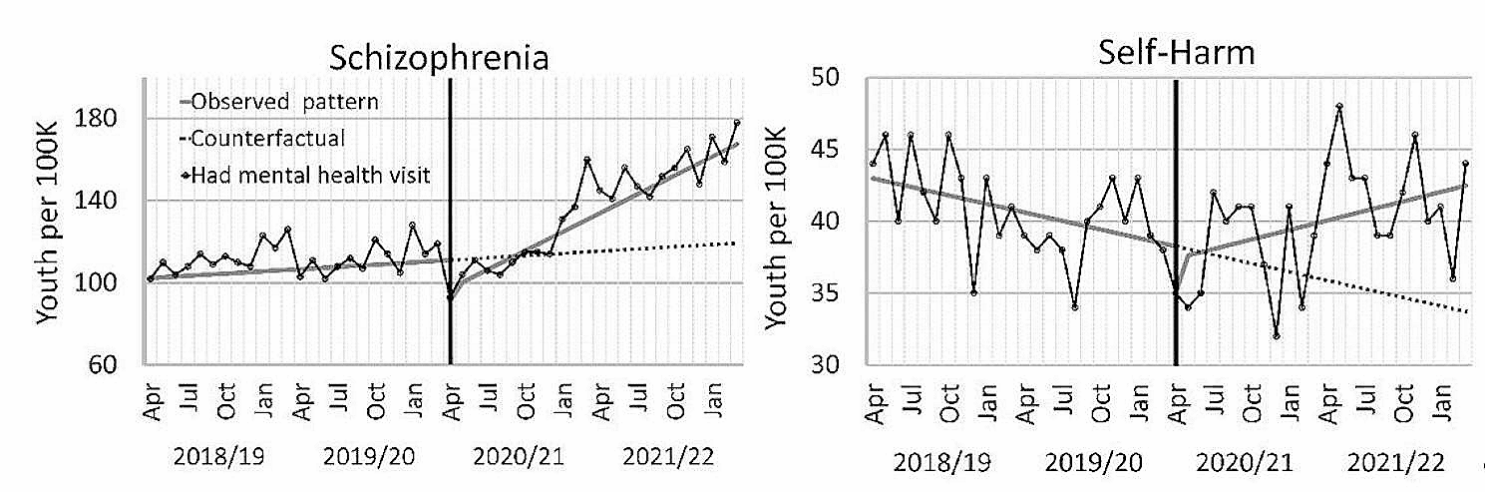
Monthly rates of Alberta youth using mental health care services for secondary diagnoses of study. The black line with points is the actual rate. Points are monthly, but labels only shown quarterly. Solid grey lines are regression lines for the observed patterns. The vertical black line marks the start of the COVID-19 pandemic. The dashed line is the continued trend from the pre-pandemic pattern (the counterfactual)

For the secondary diagnoses of study (Fig. 3), health care use for schizophrenia disorders showed a trend increase across the pandemic (+ 2.92 per month; p < 0.0001) and a delayed start to increasing in the pandemic (− 18.31; p < 0.0001). Service use for self-harm had a trend increase in the pandemic (+ 0.42 per month; p < 0.01). All other patterns were not significant. See supplement Table S5 for detailed model parameters.

As a sensitivity analysis, we investigated the type of health care use by youth for each diagnosis (see supplement Figs. S1,S2, S3, S4, S5, S6, S7). Most youth diagnoses (i.e., mood, anxiety, ADHD, substance use) were primarily seen by general physicians. Youth schizophrenia was primarily seen by psychiatrists, while youth self-harm was primarily seen in emergency room visits and hospitalizations. Youth adjustment disorder showed an even split between general physicians and psychiatrists. Overall health care use patterns followed the primary type of health care use for diagnoses. Of interest, while substance use decreased for youth seen by general physicians, it showed an increasing trend for youth seen by psychiatrists. Also, while schizophrenia diagnoses showed an increasing trend over time for youth seen by psychiatrists, it showed a drop across the pandemic for youth seen by general physicians.

### All-cause mortality

**Fig. 4:**
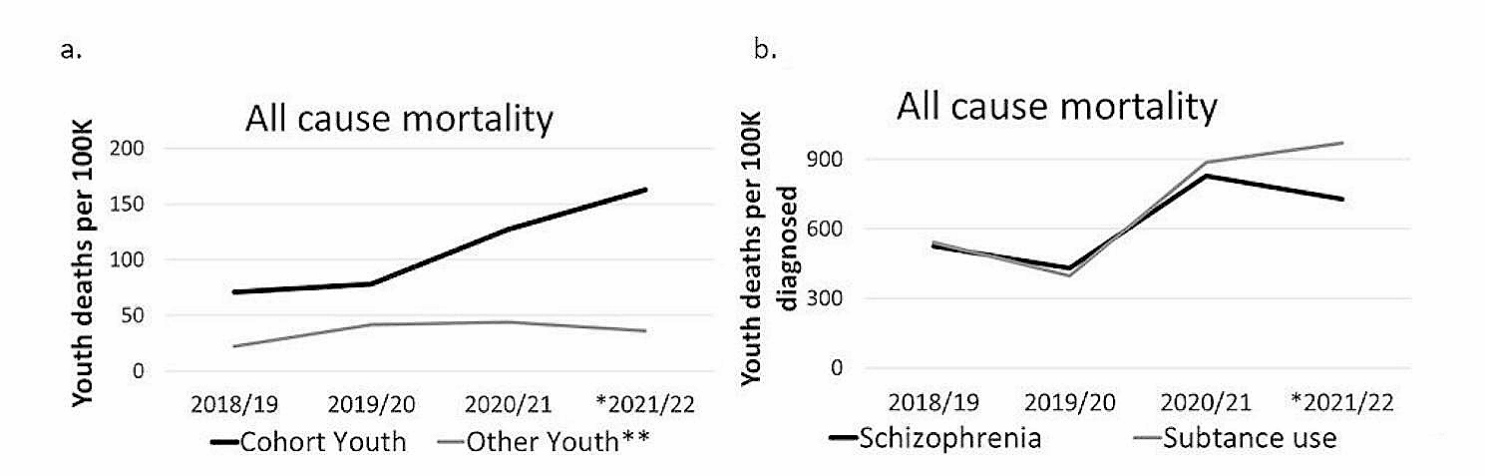
Mortality rates by presence in the cohort and for youth with a diagnosis. All cause mortality is for any reason. Diagnoses are limited within each listed fiscal year. *The 2021/22 data stops in December 2021 and rates are linearly estimated as a yearly rate. **’Other youth’ is estimated using yearly publicly reported Alberta deaths by subtracting cohort-related deaths from total deaths

All-cause youth death rates increased across the years from 71 per 100,000 youth in 2018/19 to 163 per 100,000 in 2021/22 for youth with primary or secondary mental health diagnoses, with little change seen for other youth without diagnoses (Fig. 4). Substance use and schizophrenia related disorders were the only diagnoses linked to an increase in all-cause death rates during the pandemic (Fig. 4; supplement Table S8).

### Diagnoses by youth subgroups

**Fig. 5:**
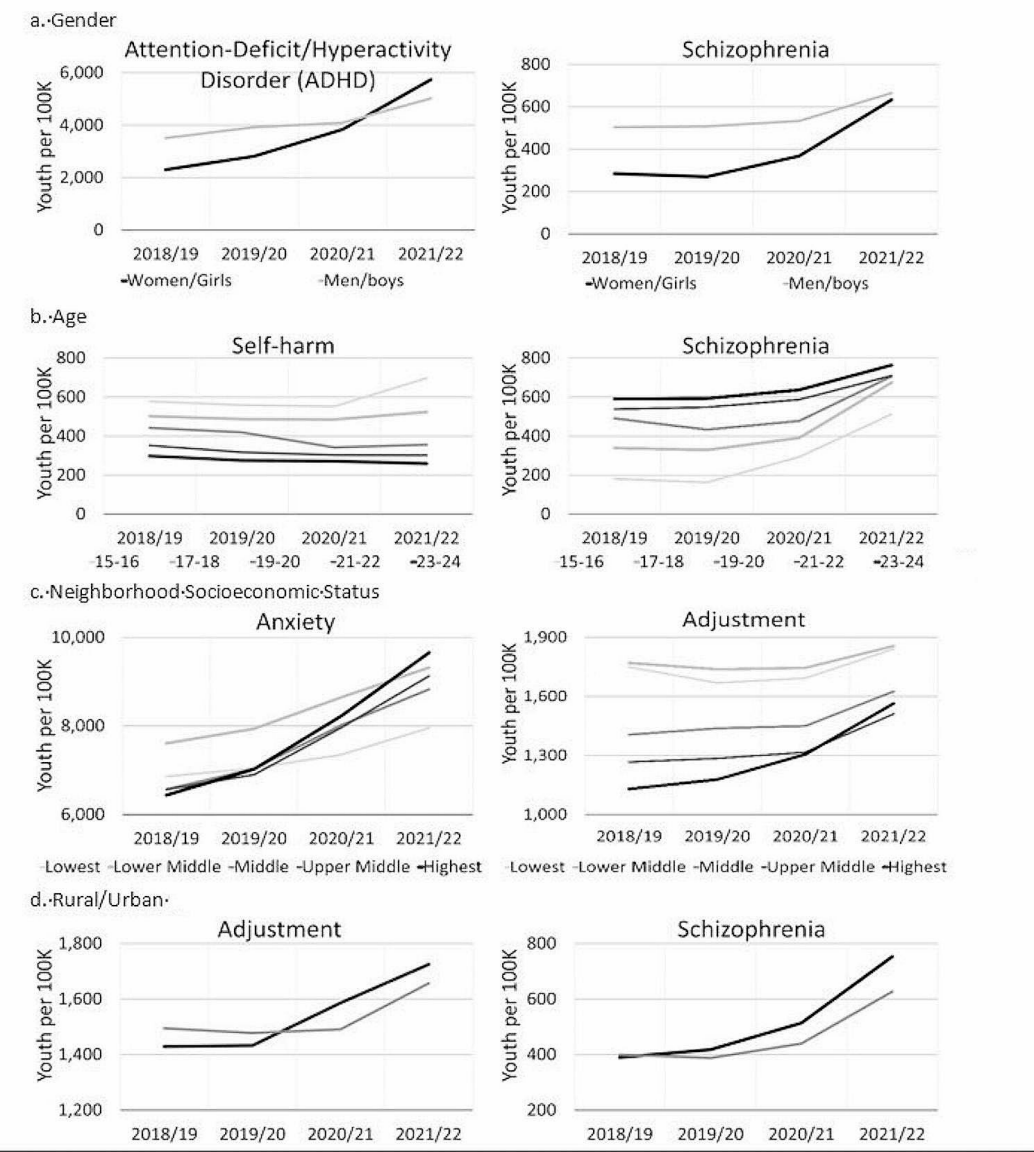
Yearly rates of Alberta youth using mental health care services for diagnoses by subgroups. Shown results are for the greatest changes in patterns over time in each characteristic

For gender, increases in service use were mostly limited to women/girls over the COVID-19 pandemic. Furthermore, diagnoses that involved greater service use for men/boys pre-pandemic (i.e., schizophrenia and ADHD) showed signs of becoming more equivalent to women/girl service use during the pandemic (Fig. 5). While most age-related diagnosis patterns prior to the pandemic held, younger youth (15–16year) showed an increase in service use for self-harm during the pandemic and younger youth (15–20year) showed a greater growth in service use for schizophrenia diagnoses than older youth (Fig. 5). Although many service use diagnosis patterns held for neighborhood socioeconomic status, youth from richer neighborhoods showed greater increases in using services for anxiety, adjustment disorder, and mood disorder over the pandemic (Fig. 5). Finally, while most diagnosis patterns held for youth rural/urban residence, greater increases in service use were seen for rural than urban youth in adjustment and schizophrenia during the pandemic (Fig. 5). See supplement Tables S9, S10, S11, S12 for full numbers.

## Discussion

### Summary

This study found an immediate drop, followed by a steady rise in mental health care use among Canadian youth in Alberta during the COVID-19 pandemic. An increase was significant for general physician (including pediatrician) and psychiatrist visits, with little change in emergency room and hospital services, which were especially overtaxed by the COVID-19 pandemic. Many diagnoses had increased service utilization trends during the pandemic, including: anxiety, adjustment, ADHD, schizophrenia, and self-harm disorders. Substance use showed an overall decrease of service use during the pandemic. Despite youth accessing services at a higher overall level during the pandemic, mortality rates greatly increased for youth being seen for mental health reasons. Youth seen for substance use and schizophrenia disorders showed the greatest increases in mortality.

Clear shifts in patterns of mental health care service use during the COVID-19 pandemic were found in this study based on social factors. First, service use increased more for women/girls. This pattern might be explained by gendered patterns of help-seeking, with women/girls often more likely than men/boys to seek mental health care support [46]. Next, youth from wealthier neighborhoods had greater health care use. Similar patterns have been seen in other research, often being explained by greater access to health care for more affluent neighborhoods [27, 28]. Finally, self-harm increases were limited to younger youth (15–16 years old). As such, the group with highest self-harm rates pre-pandemic were also most susceptible during the pandemic.

### Implications

Growing mental health challenges among Canadian youth are expected to persist into the future [1, 47], which will require ongoing access to care, supports, and resources. Our study sheds light on how health care was used for mental health challenges before and during the COVID-19 pandemic; changes in types of diagnoses over the pandemic; and the relationship between social factors and mental health care use. We discuss implications of these findings.

First, our study showed increasing mental health care use during the COVID-19 pandemic that was significant for general physician and psychiatrist visits, rather than other types of visits. Emergency room visits as a first point of contact for youth with mental health needs has been regarded as an indicator of poor access to mental health care in Canada [48]. In particular, emergency room visits are more costly to Canada’s public health care system and may involve treating mental health needs that have reached a point of crisis. Therefore, the increase in general physician visits and no increase in emergency room visits may be seen as the Albertan health care system functioning well. Other explanations for increased use of general physicians during the pandemic, however, include: discomfort in going to a potentially over-crowded emergency room; reduced public transportation to attend an in-person appointment with a specialist (e.g., psychiatrist); and the increased use of telehealth visits in Canada during the pandemic [45], which enhanced ability to access general physician services for youth. The increased use of telehealth care may have resulted in changes in quality of care [49, 50] and future studies should investigate how well these services performed during the pandemic. Ongoing research on the effects of telehealth suggests that it may have similar effectiveness to in-person care [51], although it might have drawbacks, such as higher attrition rates [52]. In addition, studies should explore how social factors interacted with the use and effectiveness of telehealth, as factors like gender and rural status are thought to differentially relate to use of telehealth [53, 54].

Second, the increased use of mental health care among Canadian youth can be interpreted as mental health needs increasing. This is consistent with other studies that have reported increased mental health needs in youth [2, 3]. While significant increases in service use were found across many of the mental health-related diagnoses in our study, an overall decrease in service use for substance use was observed. Disturbingly, this decrease was paired with an increased rate of mortality for those youth who had been seen for substance use. This finding is set in the context of a growing opioid crisis in Canada during and beyond the pandemic [55] and provides a discussion point on how well services and policies are meeting the demands of this crisis [56, 57]. This finding is corroborated by substance use surveillance findings in Alberta that showed mortality increases in recent years that were greatest for opioid-related reasons [58]. Future qualitative research on the experiences of youth and their caregivers is needed to provide context on how well needs were met for youth with substance use concerns during the pandemic.

Third, our findings show how youth differentiated by social factors accessed mental health care during the pandemic. The relationship between gender and mental health service use is broadly consistent with previous research [23], with women/girls showing greater increases in use than men/boys. These findings may suggest that women/girls were more impacted by the pandemic. However, the findings might also suggest a continued need to improve help-seeking among young men/boys to ensure that they are receiving needed services in a timely manner [23, 24]. Our results also showed a narrowing gap between genders in the use of services for ADHD which may reflect increased recognition of ADHD in women/girls by families and/or health care providers, potentially showing a reduction in diagnosis inequities. Finally, the finding of greater mental health care use by youth from more affluent neighborhoods might reflect better access to services for this group. This interpretation is in line with previously noted inequities in access to care for youth in less wealthy neighborhoods [27, 28]. This difference may have been partially driven by the shift to virtual care, which may have not been accessible financially for poorer families. Together these findings provide evidence that the COVID-19 pandemic differentially affected youth, exacerbating health inequities. Future public health emergency responses should plan for ways to mitigate inequities in mental health care access.

Fourth, physician use patterns over the pandemic present questions. Most diagnosis increases were primarily made by general physicians, except for psychiatrists for schizophrenia. This balance is not surprising in Alberta given that there were 6154 general physicians and 461 psychiatrists in 2019 [59–61]. The balance leaves more straightforward diagnoses to general physicians, with referral of more serious cases to psychiatrists. This pattern is shown in our analysis by more complicated diagnoses, such as schizophrenia and adjustment disorder, being supported by psychiatrists. While the health care system is meant to perform in this manner, it presents unique challenges in the context of the COVID-19 pandemic. As telehealth visits increased, questions present on how well general physicians were able to accurately diagnose mental health conditions by phone or virtually. Questions also remain on how well the health care system was able to triage more severe mental health needs to the limited number of psychiatrists. Future research is needed on how well severe mental health needs were supported during the pandemic.

Finally, questions remain on the trajectories of mental health care use after the COVID-19 pandemic. Will increases in ADHD diagnoses continue with self-isolation and home-based learning measures no longer in place? We have evidence that the situation surrounding the pandemic led to increased ADHD diagnoses [13], but other evidence suggests that diagnoses increase as the public becomes more aware of and accepting of mental health conditions [62]. Although it is too early to tell, increases in diagnoses in the pandemic might serve to further normalize mental health care and increase use for youth into the future. Next, rising schizophrenia diagnoses for youth may reflect earlier recognition during the pandemic. This has potential benefits to young people as early recognition and treatment have been shown to improve outcomes for young people facing psychosis [63]. In the bigger picture, general physician and psychiatrist visits were growing before the pandemic, with general physician visits accelerated by the pandemic. Such trend increases lead to questions on how well the health care system can support the growing mental health needs of young people. Future research is needed to understand the longer-term trajectories of mental health care use beyond the pandemic and how it has affected the mental health journeys of young people.

### Limitations

While this study provides novel evidence of mental health care use in Canada, it also has some key limitations. One limitation of the administrative data used for the study is its inability to capture youth who accessed services outside of the publicly funded health care system, such as private counselors or clinical psychologists, which is a significant amount of care in Alberta. As such, findings do not encompass all youth seeking mental health support, but rather a sub-population of youth engaging with the public health care system. Next, administrative data analysis has some difficulties in interpretation. For example, while increased service use may relate to increased need, it may also relate to an increased ability to navigate services. Third, the Alberta health administrative data uses a binary model of female/male sex, which limits the ability to identify inequities beyond differences between girls/women and boys/men, such as inequities among gender diverse/minority youth that have been previously documented [64]. Future work should engage with youth and families to contextualize findings and set them in real world experience. Fourth, the change from primarily in-person mental health care pre-pandemic to a blend of telehealth and in-person, and to primarily telehealth during the COVID-19 pandemic requires further exploration. This is an important factor that may have affected the quality-of-care youth received [65]. It may also relate to increased ability to access mental health care services. Future studies should investigate how youth experienced the change to telehealth services and how it affected their care. Finally, a limitation of this data is that it cannot determine the accuracy of mental health diagnoses. While the gold standard in administrative data research is prior validation of case definitions and diagnoses using patient charts [66], such validation was not possible due to a lack of standardization and centralization of patient data.

## Conclusion

This study demonstrates changes in the use of mental health care services during the COVID-19 pandemic, which differed by youth characteristics. Our findings add to growing discussion on how the pandemic affected young people. This study can inform ongoing plans to support the mental health needs of youth, future pandemic responses, and other public health emergencies.

### Supplementary material

Supplementary Material 1.

## Data Availability

The individual level data that support the findings of this study are protected and are not available due to data privacy laws. Unprocessed data is accessible for researchers through the Government of Alberta. However, the data must be housed in secure environments in Alberta, vetted through a Privacy Impact Assessment: https://www.alberta.ca/health-research. Other datasets used in this study are cited in text and are available publicly.

## Abbreviations

**ADHD**: Attention deficit/hyperactivity disorder
**SES**: Socioeconomic status
