# Supplementary material for: Youth mental health care use during the COVID-19 pandemic in Alberta, Canada: an interrupted time series, population-based study
Source: Child Adolesc Psychiatry Ment Health. 2024 Aug 10;18:101. doi: 10.1186/s13034-024-00785-1 (PMC11317004; doi:10.1186/s13034-024-00785-1)
Supplement: Supplementary file 1 — Supplementary Material 1. [file 13034_2024_785_MOESM1_ESM.pdf]

Supplement for "Youth Mental Health Care Use During COVID-19 in Alberta, Canada: An Interrupted Time Series, Cross-Sectional, Population-Based Study"

Table S1. **Diagnostic codes for primary and secondary diagnoses of study.** We used International Classification of Diseases (ICD) codes provided by physicians to match diagnoses of youth. ICD-9 was used for physician claims (general and psychiatrist), and ICD-10 was used for emergency room (ER) and hospitalization in Alberta during the study period.

**Diagnoses.**	**ICD-9 (Physician Claims)**	**ICD-10 (ER and Hospitalization)**
Mood Disorders	296, 311	F30-F39
Anxiety Disorders	300.0, 300.2, 300.3, 300.7	F40, F41, F42
Attention-Deficit/Hyperactivity Disorder (ADHD)	314	F90
Substance Use	291, 292, 303, 304, 305	F10-F19, F55
Adjustment Disorders	309	F43
Schizophrenia-related Disorders	295	F20, F21, F232, F25
Self-Harm	N/A	X60-X84, Y870

Table S2. **Percent of visits with provided diagnostic codes for each position, for a) general physician (GP) or psychiatrist claims, b) emergency room (ER) visits, and c) hospitalizations.**

**a. GP/psychiatrist**	**Percent**	**Count**
Has diagnosis 1	100.00	9,392,392
Has diagnosis 2	17.48	1,641,943
Has diagnosis 3	4.63	435,255

**b. ER**	**Percent**	**Count**
Has diagnosis 1	100.00	841,779
Has diagnosis 2	53.91	453,823
Has diagnosis 3	34.01	286,293
Has diagnosis 4	10.92	91,926
Has diagnosis 5	4.42	37,218
Has diagnosis 6	2.16	18,172
Has diagnosis 7	1.18	9,935
Has diagnosis 8	0.67	5,666
Has diagnosis 9	0.38	3,193
Has diagnosis 10	0.21	1,742

**c. Hospitalization**	**Percent**	**Count**
Has diagnosis 1	100.00	89,721
Has diagnosis 2	78.78	70,682
Has diagnosis 3	57.52	51,605
Has diagnosis 4	37.82	33,936
Has diagnosis 5	23.79	21,346
Has diagnosis 6	14.77	13,250
Has diagnosis 7	9.39	8,428
Has diagnosis 8	6.08	5,459
Has diagnosis 9	4.02	3,605
Has diagnosis 10	2.68	2,408
Has diagnosis 11	1.83	1,639
Has diagnosis 12	1.30	1,168
Has diagnosis 13	0.95	853
Has diagnosis 14	0.73	655
Has diagnosis 15	0.56	506
Has diagnosis 16	0.45	402
Has diagnosis 17	0.35	318
Has diagnosis 18	0.27	246
Has diagnosis 19	0.23	202
Has diagnosis 20	0.19	166
Has diagnosis 21	0.14	128
Has diagnosis 22	0.13	113
Has diagnosis 23	0.10	94
Has diagnosis 24	0.08	76
Has diagnosis 25	0.07	65

Table S3. Monthly number and rate per 100,000 youth of youth using mental health care services, based on the most responsible diagnosis during visits.

	**2018/19**											
**Mental Health Visits**	**4**	**5**	**6**	**7**	**8**	**9**	**10**	**11**	**12**	**1**	**2**	**3**
General Physician Use #	12,775	12,818	12,234	11,571	12,391	12,174	14,235	14,033	12,284	14,746	12,874	14,320
General Physician Rate/100K	2,445	2,451	2,337	2,209	2,363	2,320	2,710	2,670	2,335	2,801	2,443	2,715
Psychiatrist Use #	4,635	4,934	4,709	4,307	4,423	4,402	4,956	4,921	4,175	4,941	4,779	5,007
Psychiatrist Rate/100K	887	943	900	822	844	839	944	936	794	938	907	949
Emergency Room Use #	1,510	1,568	1,540	1,509	1,445	1,459	1,613	1,534	1,475	1,438	1,249	1,512
Emergency Room Rate/100K	289	300	294	288	276	278	307	292	280	273	237	287
Hospitalization Use #	352	376	342	361	343	320	406	373	361	395	306	361
Hospitalization Rate/100K	67	72	65	69	65	61	77	71	69	75	58	68
Youth Population #	522,549	522,992	523,436	523,879	524,323	524,766	525,209	525,653	526,096	526,540	526,983	527,427

	**2019/20**											
**Mental Health Visits**	**4**	**5**	**6**	**7**	**8**	**9**	**10**	**11**	**12**	**1**	**2**	**3**
General Physician Use #	13,530	13,948	12,439	12,762	12,720	13,330	15,121	14,585	13,678	15,561	14,613	14,920
General Physician Rate/100K	2,563	2,640	2,352	2,412	2,402	2,515	2,851	2,749	2,577	2,930	2,750	2,807
Psychiatrist Use #	4,932	5,016	4,726	4,645	4,556	4,687	5,259	5,035	4,404	5,093	4,854	5,154
Psychiatrist Rate/100K	934	949	894	878	860	884	992	949	830	959	914	970
Emergency Room Use #	1,307	1,392	1,431	1,383	1,246	1,449	1,569	1,475	1,389	1,454	1,452	1,267
Emergency Room Rate/100K	248	263	271	261	235	273	296	278	262	274	273	238
Hospitalization Use #	295	312	328	314	303	362	401	379	327	365	355	356
Hospitalization Rate/100K	56	59	62	59	57	68	76	71	62	69	67	67
Youth Population #	527,870	528,313	528,757	529,200	529,644	530,087	530,341	530,596	530,850	531,105	531,359	531,614

	**2020/21**											
**Mental Health Visits**	**4**	**5**	**6**	**7**	**8**	**9**	**10**	**11**	**12**	**1**	**2**	**3**
General Physician Use #	11,748	12,581	14,455	14,605	14,069	16,043	16,905	17,193	16,599	17,921	17,273	20,401
General Physician Rate/100K	2,209	2,364	2,715	2,742	2,640	3,009	3,172	3,227	3,116	3,365	3,244	3,833
Psychiatrist Use #	4,941	5,010	5,345	5,034	4,863	5,259	5,478	5,590	5,197	5,711	5,551	6,194
Psychiatrist Rate/100K	929	942	1,004	945	913	986	1,028	1,049	976	1,072	1,043	1,164
Emergency Room Use #	970	1276	1401	1457	1363	1336	1419	1271	1176	1304	1275	1432
Emergency Room Rate/100K	182	240	263	274	256	251	266	239	221	245	239	269
Hospitalization Use #	283	326	377	360	347	361	406	324	310	341	393	401
Hospitalization Rate/100K	53	61	71	68	65	68	76	61	58	64	74	75
Youth Population #	531,868	532,122	532,377	532,631	532,886	533,140	532,995	532,850	532,706	532,561	532,416	532,271

	**2021/22**											
**Mental Health Visits**	**4**	**5**	**6**	**7**	**8**	**9**	**10**	**11**	**12**	**1**	**2**	**3**
General Physician Use #	18,330	17,838	18,451	16,098	17,036	18,244	18,885	20,156	18,297	19,261	18,271	20,747
General Physician Rate/100K	3,445	3,353	3,469	3,028	3,205	3,433	3,544	3,771	3,414	3,584	3,390	3,838
Psychiatrist Use #	5,925	5,821	6,067	5,193	5,097	5,481	5,797	5,938	5,190	5,921	5,378	6,149
Psychiatrist Rate/100K	1,113	1,094	1,141	977	959	1,031	1,088	1,111	968	1,102	998	1,138
Emergency Room Use #	1,327	1,438	1,514	1,435	1,452	1,235	1,480	1,465	1,315	1,255	1,158	1,365
Emergency Room Rate/100K	249	270	285	270	273	232	278	274	245	233	215	253
Hospitalization Use #	384	378	361	340	310	315	410	410	321	381	330	345
Hospitalization Rate/100K	72	71	68	64	58	59	77	77	60	71	61	64
Youth Population #	532,126	531,981	531,837	531,692	531,547	531,402	532,924	534,446	535,968	537,490	539,012	540,534

*General physician claims include general and family physicians, and pediatricians.

Table S4. Parameters for interrupted time series, segmented regression models performed on monthly rates of use per 100,000 youth for each type of mental health care service use. Pre-pandemic trend represents the slope before the pandemic, pandemic start is the first month in the pandemic, pandemic the correction across the pandemic, and pandemic trend the change in slope over the pandemic.

General Physician Claims

Variable	Estimate	Standard Error	p-value
Intercept	2403	79.33	<.0001
Monthly trend	11.75	4.79	0.018
Pandemic start	-657.35	177.48	0.001
Pandemic	67.91	87.86	0.444
Pandemic trend	27.24	7.21	0.001

**Autocorrelation found and corrected for.*

Psychiatrist Claims

Variable	Estimate	Standard Error	p-value
Intercept	882.4508	21.3808	<.0001
Monthly trend	1.6788	1.0658	0.123
Pandemic start	-106.095	37.5993	0.007
Pandemic	58.0354	18.2913	0.003
Pandemic trend	2.6543	1.5813	0.101

**Autocorrelation found and corrected for.*

Emergency Room

Variable	Estimate	Standard Error	p-value
Intercept	291.75	7.76	<.0001
Monthly trend	-1.43	0.54	0.012
Pandemic start	-74.52	20.05	0.001
Pandemic	-0.69	11.16	0.951
Pandemic trend	1.21	0.79	0.134

Hospitalization

Variable	Estimate	Standard Error	p-value
Intercept	67.51	2.65	<.0001
Monthly trend	-0.1	0.19	0.589
Pandemic start	-14.53	6.84	0.040
Pandemic	2.47	3.81	0.520
Pandemic trend	0.06	0.27	0.813

Table S5. Parameters for interrupted time series, segmented regression models performed on monthly rates of use per 100,000 youth for each mental health care diagnosis. Pre-pandemic trend represents the slope before the pandemic, pandemic start is the first month in the pandemic, pandemic the correction across the pandemic, and pandemic trend the change in slope over the pandemic.

Mood

Variable	Estimate	Standard Error	p-value
Intercept	1475	60.15	<.0001
Pre-pandemic trend	1.33	2.69	0.624
Pandemic start	-321.53	80.67	<0.001
Pandemic	95.79	45.36	0.041
Pandemic trend	3.13	3.95	0.433

**Autocorrelation found and corrected for.*

Anxiety

Variable	Estimate	Standard Error	p-value
Intercept	959.86	36.36	<.0001
Pre-pandemic trend	7.17	2.54	0.007
Pandemic start	-256.59	93.97	0.009
Pandemic	90.08	52.29	0.092
Pandemic trend	7.45	3.72	0.052

ADHD

Variable	Estimate	Standard Error	p-value
Intercept	466.72	16.21	<.0001
Pre-pandemic trend	6.85	0.97	<.0001
Pandemic start	-91.89	38.45	0.022
Pandemic	-46.08	17.57	0.012
Pandemic trend	15.80	1.46	<.0001

**Autocorrelation found and corrected for.*

Substance Use

Variable	Estimate	Standard Error	p-value
Intercept	328.81	6.85	<.0001
Pre-pandemic trend	-0.51	0.48	0.292
Pandemic start	-48.51	17.71	0.009
Pandemic	-34.65	9.85	0.001
Pandemic trend	1.13	0.70	0.114

Adjustment Disorder

Variable	Estimate	Standard Error	p-value
Intercept	178.81	4.91	<.0001
Pre-pandemic trend	-0.16	0.36	0.659
Pandemic start	-45.89	12.01	0.001
Pandemic	6.78	7.80	0.390
Pandemic trend	1.87	0.48	<0.001

**Autocorrelation found and corrected for.*

Schizophrenia

Variable	Estimate	Standard Error	p-value
Intercept	108.56	2.77	<.0001
Pre-pandemic trend	0.27	0.20	0.188
Pandemic start	-5.69	7.82	0.471
Pandemic	-18.31	4.23	<.0001
Pandemic trend	2.92	0.28	<.0001

**Autocorrelation found and corrected for.*

Self-harm

Variable	Estimate	Standard Error	p-value
Intercept	43.17	1.42	<.0001
Pre-pandemic trend	-0.20	0.10	0.054
Pandemic start	-2.40	3.67	0.517
Pandemic	-1.26	2.04	0.539
Pandemic trend	0.42	0.15	0.006

Table S6. Monthly numbers and rates of youth using health care services for primary diagnoses of study.

	2018/19											
**Mental Health Visits**	**4**	**5**	**6**	**7**	**8**	**9**	**10**	**11**	**12**	**1**	**2**	**3**
Mood #	7,547	7,810	7,574	6,982	7,152	7,158	8,399	8,477	7,269	8,677	7,822	8,650
Rate/100k	1,444	1,493	1,447	1,333	1,364	1,364	1,599	1,613	1,382	1,648	1,484	1,640
Anxiety #	5,248	5,317	5,020	4,842	5,113	5,059	5,927	5,623	4,889	5,809	5,334	5,751
Rate/100k	1,004	1,017	959	924	975	964	1,129	1,070	929	1,103	1,012	1,090
ADHD #	2,809	2,766	2,499	2,120	2,580	2,553	2,929	2,876	2,520	3,047	2,597	2,862
Rate/100k	538	529	477	405	492	487	558	547	479	579	493	543
Substance Use #	1,698	1,699	1,686	1,715	1,756	1,640	1,844	1,792	1,690	1,868	1,631	1,822
Rate/100k	325	325	322	327	335	313	351	341	321	355	309	345
Adjustment #	948	975	970	909	951	908	1,007	956	828	949	825	942
Rate/100k	181	186	185	174	181	173	192	182	157	180	157	179
Youth Population #	522,549	522,992	523,436	523,879	524,323	524,766	525,209	525,653	526,096	526,540	526,983	527,427

	2019/20											
**Mental Health Visits**	**4**	**5**	**6**	**7**	**8**	**9**	**10**	**11**	**12**	**1**	**2**	**3**
Mood #	7,947	8,098	7,363	7,589	7,248	7,444	8,646	8,367	7,658	8,806	8,290	8,448
Rate/100k	1,505	1,533	1,393	1,434	1,368	1,404	1,630	1,577	1,443	1,658	1,560	1,589
Anxiety #	5,548	5,785	5,167	5,317	5,230	5,479	6,206	6,081	5,487	6,329	6,067	6,260
Rate/100k	1,051	1,095	977	1,005	987	1,034	1,170	1,146	1,034	1,192	1,142	1,178
ADHD #	3,130	3,266	2,788	2,668	2,879	3,121	3,556	3,358	3,102	3,501	3,235	3,436
Rate/100k	593	618	527	504	544	589	671	633	584	659	609	646
Substance Use #	1,554	1,574	1,572	1,648	1,598	1,725	1,749	1,672	1,643	1,806	1,728	1,712
Rate/100k	294	298	297	311	302	325	330	315	310	340	325	322
Adjustment #	933	930	953	884	882	928	1,086	1,030	860	977	935	911
Rate/100k	177	176	180	167	167	175	205	194	162	184	176	171
Youth Population #	527,870	528,313	528,757	529,200	529,644	530,087	530,341	530,596	530,850	531,105	531,359	531,614

	2020/21											
**Mental Health Visits**	**4**	**5**	**6**	**7**	**8**	**9**	**10**	**11**	**12**	**1**	**2**	**3**
Mood #	7,108	7,488	8,308	8,199	7,722	8,436	8,970	9,211	8,727	9,541	9,189	10,499
Rate/100k	1,336	1,407	1,561	1,539	1,449	1,582	1,683	1,729	1,638	1,792	1,726	1,972
Anxiety #	5,212	5,697	6,462	6,552	6,398	7,048	7,308	7,414	7,279	7,861	7,540	8,876
Rate/100k	980	1,071	1,214	1,230	1,201	1,322	1,371	1,391	1,366	1,476	1,416	1,668
ADHD #	2989	2993	3336	3169	3249	4126	4349	4420	4120	4386	4263	5104
Rate/100k	562	562	627	595	610	774	816	830	773	824	801	959
Substance Use #	1246	1404	1614	1565	1513	1525	1551	1466	1493	1569	1579	1734
Rate/100k	234	264	303	294	284	286	291	275	280	295	297	326
Adjustment #	732	874	983	1,007	893	1,028	1,090	1,100	959	1,065	1,043	1,237
Rate/100k	138	164	185	189	168	193	205	206	180	200	196	232
Youth Population #	531,868	532,122	532,377	532,631	532,886	533,140	532,995	532,850	532,706	532,561	532,416	532,271

	2021/22											
**Mental Health Visits**	**4**	**5**	**6**	**7**	**8**	**9**	**10**	**11**	**12**	**1**	**2**	**3**
Mood #	9,377	9,157	9,472	8,174	8,261	8,490	8,883	9,509	8,591	9,336	8,564	9,942
Rate/100k	1,762	1,721	1,781	1,537	1,554	1,598	1,667	1,779	1,603	1,737	1,589	1,839
Anxiety #	8,084	7,786	8,083	7,067	7,301	8,021	8,092	8,605	7,617	8,064	7,574	8,548
Rate/100k	1,519	1,464	1,520	1,329	1,374	1,509	1,518	1,610	1,421	1,500	1,405	1,581
ADHD #	5,042	4,966	5,129	4,218	4,779	5,408	5,712	6,037	5,389	5,732	5,542	6,269
Rate/100k	948	933	964	793	899	1,018	1,072	1,130	1,005	1,066	1,028	1,160
Substance Use #	1,441	1,444	1,538	1,521	1,578	1,442	1,586	1,587	1,511	1,591	1,537	1,790
Rate/100k	271	271	289	286	297	271	298	297	282	296	285	331
Adjustment #	1,125	1,172	1,170	1,108	1,042	1,048	1,258	1,240	1,092	1,104	1,079	1,198
Rate/100k	211	220	220	208	196	197	236	232	204	205	200	222
Youth Population #	532,126	531,981	531,837	531,692	531,547	531,402	532,924	534,446	535,968	537,490	539,012	540,534

Table S7. Monthly numbers and rates of youth using health care services for secondary diagnoses of study.

	2018/19											
**Mental Health Visits**	**4**	**5**	**6**	**7**	**8**	**9**	**10**	**11**	**12**	**1**	**2**	**3**
Schizophrenia #	532	577	542	566	597	571	596	580	566	649	617	662
Rate/100k	102	110	104	108	114	109	113	110	108	123	117	126
Self-Harm #	229	242	207	239	219	208	239	224	185	226	207	218
Rate/100k	44	46	40	46	42	40	46	43	35	43	39	41
Youth Population #	522,549	522,992	523,436	523,879	524,323	524,766	525,209	525,653	526,096	526,540	526,983	527,427

	2019/20											
**Mental Health Visits**	**4**	**5**	**6**	**7**	**8**	**9**	**10**	**11**	**12**	**1**	**2**	**3**
Schizophrenia #	546	585	537	571	591	568	643	606	558	682	605	634
Rate/100k	103	111	102	108	112	107	121	114	105	128	114	119
Self-Harm #	205	203	206	202	179	214	219	230	213	226	205	200
Rate/100k	39	38	39	38	34	40	41	43	40	43	39	38
Youth Population #	527,870	528,313	528,757	529,200	529,644	530,087	530,341	530,596	530,850	531,105	531,359	531,614

	2020/21											
**Mental Health Visits**	**4**	**5**	**6**	**7**	**8**	**9**	**10**	**11**	**12**	**1**	**2**	**3**
Schizophrenia #	497	555	591	567	552	587	611	614	609	695	728	853
Rate/100k	93	104	111	106	104	110	115	115	114	131	137	160
Self-Harm #	186	180	185	225	211	217	221	199	173	221	183	207
Rate/100k	35	34	35	42	40	41	41	37	32	41	34	39
Youth Population #	531,868	532,122	532,377	532,631	532,886	533,140	532,995	532,850	532,706	532,561	532,416	532,271

	2021/22											
**Mental Health Visits**	**4**	**5**	**6**	**7**	**8**	**9**	**10**	**11**	**12**	**1**	**2**	**3**
Schizophrenia #	774	752	831	783	753	808	829	880	793	921	855	961
Rate/100k	145	141	156	147	142	152	156	165	148	171	159	178
Self-Harm #	233	257	227	230	206	209	222	246	216	220	193	238
Rate/100k	44	48	43	43	39	39	42	46	40	41	36	44
Youth Population #	532,126	531,981	531,837	531,692	531,547	531,402	532,924	534,446	535,968	537,490	539,012	540,534

Table S8. Yearly all cause mortality numbers and rates a) for youth with a most responsible diagnosis in the year (rate based on number of youth with diagnoses) and d) by youth's presence in the cohort (rate based on the Alberta youth population).

a. All cause deaths	2018/19	2019/20	2020/21	*2021/22
Mood #	65	59	58	58
Yearly diagnoses	40,675	41,896	43,158	46,062
Yearly rate/100k diagnoses	160	141	134	168
Anxiety #	42	18	37	26
Yearly diagnoses	36,215	38,232	42,816	47,826
Yearly rate/100k diagnoses	116	47	86	72
ADHD #	12	8	12	5
Yearly diagnoses	15,401	17,972	21,152	28,766
Yearly rate/100k diagnoses	78	45	57	23
Substance use #	57	39	77	62
Yearly diagnoses	10,508	9,812	8,672	8,524
Yearly rate/100k diagnoses	542	397	888	970
Adjustment #	24	17	24	19
Yearly diagnoses	7,806	7,802	8,030	8,947
Yearly rate/100k diagnoses	307	218	299	283
Schizophrenia #	11	9	20	19
Yearly diagnoses	2,095	2,087	2,414	3,481
Yearly rate/100k diagnoses	525	431	829	728
Self-Harm #	16	21	17	14
Yearly diagnoses	2,260	2,160	2,061	2,271
Yearly rate/100k diagnoses	708	972	825	822

b. All cause deaths	2018/19	2019/20	2020/21	*2021/22
Mental health cohort	116	133	217	204
Youth population	163,774	170,139	171,424	166,920
Yearly rate/100k youth	71	78	127	163
Other youth**	80	151	160	132
Youth population	362,322	360,712	361,281	369,048
Yearly rate/100k youth	22	42	44	36

*2021/22 health care data ends in December 2021. Rates for this year are estimated linearly for the year.
**'Other youth' is estimated using yearly Alberta deaths by subtracting cohort deaths from total deaths.

Table S9. **Gender, and yearly numbers and rates of Alberta youth using mental health care services for each most responsible diagnosis.**

Women/girls	2018/19	2019/20	2020/21	2021/22
Mood #	25,771	26,540	28,188	30,693
Rate/100k	10,127	10,325	10,921	11,799
Anxiety #	23,883	25,386	29,393	33,126
Rate/100k	9,385	9,876	11,388	12,734
ADHD #	5,867	7,233	9,907	14,922
Rate/100k	2,306	2,814	3,838	5,736
Substance use #	4,798	4,532	3,903	4,061
Rate/100k	1,885	1,763	1,512	1,561
Adjustment #	4,845	4,880	5,070	5,882
Rate/100k	1,904	1,898	1,964	2,261
Schizophrenia #	726	695	950	1,647
Rate/100k	285	270	368	633
Self-Harm #	1,592	1,474	1,447	1,687
Rate/100k	626	573	561	649
Youth Population #	254,469	257,051	258,099	260,131

Men/boys	2018/19	2019/20	2020/21	2021/22
Mood #	14,903	15,355	14,969	15,369
Rate/100k	5,487	5,608	5,451	5,572
Anxiety #	12,331	12,846	13,423	14,700
Rate/100k	4,540	4,692	4,888	5,329
ADHD #	9,534	10,739	11,245	13,844
Rate/100k	3,510	3,922	4,095	5,019
Substance use #	5,710	5,280	4,769	4,463
Rate/100k	2,102	1,928	1,737	1,618
Adjustment #	2,961	2,922	2,960	3,065
Rate/100k	1,090	1,067	1,078	1,111
Schizophrenia #	1,369	1,392	1,464	1,834
Rate/100k	504	508	533	665
Self-Harm #	668	686	614	584
Rate/100k	246	251	224	212
Youth Population #	271,628	273,800	274,606	275,837

Table S10. **Age, and yearly numbers and rates of Alberta youth using mental health care services for each most responsible diagnosis.**

Age 15-16 in 2020/21	2018/19	2019/20	2020/21	2021/22
Mood #	7,570	7,975	8,574	9,832
Rate/100k	7,665	7,862	8,340	9,389
Anxiety #	7,264	8,132	9,023	10,868
Rate/100k	7,355	8,017	8,777	10,378
ADHD #	5,021	5,993	6,679	8,545
Rate/100k	5,084	5,908	6,497	8,160
Substance use #	2,139	2,042	1,853	1,922
Rate/100k	2,166	2,013	1,802	1,835
Adjustment #	2,166	2,080	2,100	2,443
Rate/100k	2,193	2,051	2,043	2,333
Schizophrenia #	179	165	301	536
Rate/100k	181	163	293	512
Self-Harm #	571	567	568	730
Rate/100k	578	559	552	697
Youth Population #	98,765	101,432	102,808	104,717

Age 17-18 in 2020/21	2018/19	2019/20	2020/21	2021/22
Mood #	9,116	9,078	9,846	10,992
Rate/100k	9,057	9,023	9,694	10,614
Anxiety #	8,146	8,603	9,818	11,550
Rate/100k	8,094	8,551	9,667	11,153
ADHD #	3,970	4,539	5,309	7,112
Rate/100k	3,945	4,511	5,227	6,867
Substance use #	3,154	2,903	2,643	2,659
Rate/100k	3,134	2,885	2,602	2,567
Adjustment #	2,025	2,004	2,050	2,329
Rate/100k	2,012	1,992	2,018	2,249
Schizophrenia #	341	331	397	698
Rate/100k	339	329	391	674
Self-Harm #	505	490	493	543
Rate/100k	502	487	485	524
Youth Population #	100,646	100,612	101,564	103,564

Age 19-20 in 2020/21	2018/19	2019/20	2020/21	2021/22
Mood #	10,106	10,472	10,767	11,335
Rate/100k	9,589	9,791	10,124	10,778
Anxiety #	9,068	9,828	10,714	11,564
Rate/100k	8,604	9,189	10,074	10,996
ADHD #	3,252	3,794	4,652	6,278
Rate/100k	3,086	3,547	4,374	5,969
Substance use #	3,863	3,502	3,112	3,039
Rate/100k	3,665	3,274	2,926	2,890
Adjustment #	1,958	2,039	2,119	2,196
Rate/100k	1,858	1,906	1,992	2,088
Schizophrenia #	517	463	508	742
Rate/100k	491	433	478	706
Self-Harm #	466	448	364	374
Rate/100k	442	419	342	356
Youth Population #	105,394	106,953	106,353	105,168

Age 21-22 in 2020/21	2018/19	2019/20	2020/21	2021/22
Mood #	10,357	10,773	11,281	11,995
Rate/100k	9,631	9,949	10,318	10,877
Anxiety #	9,372	9,759	11,083	12,115
Rate/100k	8,715	9,012	10,137	10,986
ADHD #	2,946	3,502	4,335	5,976
Rate/100k	2,740	3,234	3,965	5,419
Substance use #	4,256	3,917	3,602	3,461
Rate/100k	3,958	3,617	3,294	3,138
Adjustment #	1,939	1,963	2,141	2,321
Rate/100k	1,803	1,813	1,958	2,105
Schizophrenia #	579	593	642	783
Rate/100k	538	548	587	710
Self-Harm #	379	343	331	333
Rate/100k	352	317	303	302
Youth Population #	107,533	108,283	109,335	110,278

Age 23-24 in 2020/21	2018/19	2019/20	2020/21	2021/22
Mood #	10,740	11,373	11,891	12,310
Rate/100k	9,441	10,014	10,556	10,967
Anxiety #	9,955	10,380	11,737	12,386
Rate/100k	8,751	9,140	10,419	11,035
ADHD #	2,672	3,142	3,979	5,551
Rate/100k	2,349	2,767	3,532	4,946
Substance use #	4,874	4,539	4,099	3,746
Rate/100k	4,284	3,997	3,639	3,337
Adjustment #	2,111	2,145	2,252	2,343
Rate/100k	1,856	1,889	1,999	2,087
Schizophrenia #	671	674	718	857
Rate/100k	590	593	637	764
Self-Harm #	339	312	305	291
Rate/100k	298	275	271	259
Youth Population #	113,759	113,571	112,646	112,241

Table S11. **Neighborhood socioeconomic status (SES), and yearly numbers and rates of Alberta youth using mental health care services for each most responsible diagnosis.**

Lowest SES	2018/19	2019/20	2020/21	2021/22
Mood #	8,963	8,916	8,877	9,210
Rate/100k	8,603	8,548	8,501	8,826
Anxiety #	7,149	7,346	7,678	8,300
Rate/100k	6,862	7,043	7,353	7,954
ADHD #	2,450	2,687	3,066	4,303
Rate/100k	2,352	2,576	2,936	4,123
Substance use #	3,306	3,051	2,708	2,512
Rate/100k	3,173	2,925	2,593	2,407
Adjustment #	1,822	1,741	1,768	1,921
Rate/100k	1,749	1,669	1,693	1,841
Schizophrenia #	607	604	668	912
Rate/100k	583	579	640	874
Self-Harm #	650	632	600	615
Rate/100k	624	606	575	589
Youth Population #	104,179	104,300	104,421	104,356

Upper Middle SES	2018/19	2019/20	2020/21	2021/22
Mood #	7,219	7,472	7,753	8,618
Rate/100k	6,918	7,152	7,413	8,245
Anxiety #	6,851	7,209	8,330	9,541
Rate/100k	6,565	6,900	7,964	9,128
ADHD #	3,154	3,709	4,384	6,089
Rate/100k	3,023	3,550	4,192	5,825
Substance use #	1,340	1,295	1,117	1,177
Rate/100k	1,284	1,240	1,068	1,126
Adjustment #	1,322	1,342	1,377	1,579
Rate/100k	1,267	1,285	1,317	1,511
Schizophrenia #	300	281	340	523
Rate/100k	287	269	325	500
Self-Harm #	333	306	312	342
Rate/100k	319	293	298	327
Youth Population #	104,350	104,471	104,592	104,527

Lower Middle SES	2018/19	2019/20	2020/21	2021/22
Mood #	9,638	9,790	9,840	10,126
Rate/100k	9,094	9,227	9,263	9,538
Anxiety #	8,064	8,421	9,188	9,892
Rate/100k	7,609	7,937	8,649	9,318
ADHD #	2,929	3,471	3,995	5,353
Rate/100k	2,764	3,271	3,761	5,042
Substance use #	2,696	2,468	2,156	2,118
Rate/100k	2,544	2,326	2,030	1,995
Adjustment #	1,876	1,844	1,854	1,970
Rate/100k	1,770	1,738	1,745	1,856
Schizophrenia #	542	540	607	847
Rate/100k	511	509	571	798
Self-Harm #	556	524	491	517
Rate/100k	525	494	462	487
Youth Population #	105,981	106,104	106,226	106,161

Highest SES	2018/19	2019/20	2020/21	2021/22
Mood #	6,403	6,857	7,409	8,278
Rate/100k	6,220	6,654	7,181	8,028
Anxiety #	6,628	7,239	8,490	9,955
Rate/100k	6,439	7,024	8,229	9,655
ADHD #	3,828	4,552	5,371	7,205
Rate/100k	3,719	4,417	5,206	6,988
Substance use #	1,090	969	871	988
Rate/100k	1,059	940	844	958
Adjustment #	1,163	1,214	1,347	1,613
Rate/100k	1,130	1,178	1,306	1,564
Schizophrenia #	223	234	282	462
Rate/100k	217	227	273	448
Self-Harm #	237	247	241	298
Rate/100k	230	240	234	289
Youth Population #	102,938	103,057	103,176	103,112

Middle SES	2018/19	2019/20	2020/21	2021/22
Mood #	7,723	8,131	8,527	8,970
Rate/100k	7,388	7,769	8,138	8,566
Anxiety #	6,868	7,362	8,402	9,243
Rate/100k	6,570	7,034	8,019	8,827
ADHD #	2,791	3,283	4,007	5,328
Rate/100k	2,670	3,137	3,824	5,088
Substance use #	1,796	1,755	1,550	1,459
Rate/100k	1,718	1,677	1,479	1,393
Adjustment #	1,470	1,505	1,519	1,703
Rate/100k	1,406	1,438	1,450	1,626
Schizophrenia #	382	384	463	665
Rate/100k	365	367	442	635
Self-Harm #	424	389	359	434
Rate/100k	406	372	343	414
Youth Population #	104,536	104,658	104,779	104,714

Table S12. Rural/urban residence, and yearly numbers and rates of Alberta youth using mental health care services for each most responsible diagnosis.

Rural	2018/19	2019/20	2020/21	2021/22
Mood #	7,288	7,716	8,029	8,584
Rate/100k	7,816	8,245	8,567	9,094
Anxiety #	6,311	6,888	7,382	8,095
Rate/100k	6,768	7,360	7,876	8,576
ADHD #	1,836	2,090	2,498	3,711
Rate/100k	1,969	2,233	2,665	3,931
Substance use #	2,562	2,458	2,353	2,271
Rate/100k	2,747	2,626	2,511	2,406
Adjustment #	1,333	1,341	1,486	1,628
Rate/100k	1,429	1,433	1,586	1,725
Schizophrenia #	364	391	482	711
Rate/100k	390	418	514	753
Self-Harm #	554	537	537	556
Rate/100k	594	574	573	589
Youth Population #	93,250	93,588	93,722	94,394

Urban	2018/19	2019/20	2020/21	2021/22
Mood #	33,387	34,180	35,129	37,478
Rate/100k	7,713	7,817	8,002	8,487
Anxiety #	29,904	31,344	35,434	39,731
Rate/100k	6,909	7,168	8,072	8,998
ADHD #	13,565	15,882	18,654	25,055
Rate/100k	3,134	3,632	4,249	5,674
Substance use #	7,946	7,354	6,319	6,253
Rate/100k	1,836	1,682	1,439	1,416
Adjustment #	6,473	6,461	6,544	7,319
Rate/100k	1,495	1,478	1,491	1,657
Schizophrenia #	1,731	1,696	1,932	2,770
Rate/100k	400	388	440	627
Self-Harm #	1,706	1,623	1,524	1,715
Rate/100k	394	371	347	388
Youth Population #	432,846	437,262	438,984	441,574

Figure 1. **Rates of mental health care use split by all mental health care, general physician, and psychiatrist visits, for mood disorders.**

a. All mental health care visits

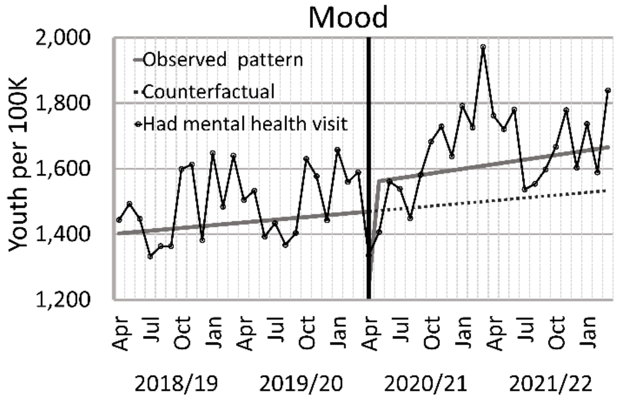

b. General physician visits

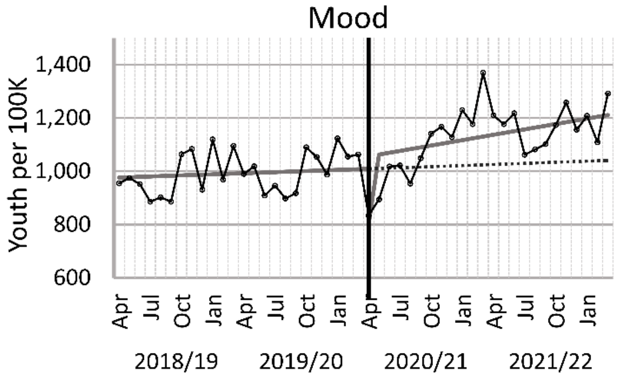

c. Psychiatrist visits

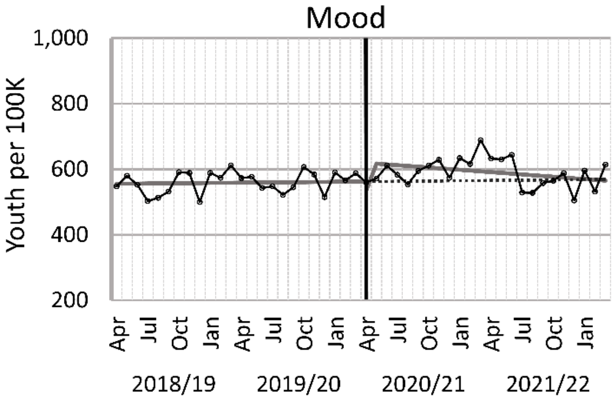

The black line with points is the actual rate. Points are monthly, but labels only shown quarterly. Solid grey lines are regression lines for the observed patterns. The vertical black line marks the start of the COVID-19 pandemic. The dashed line is the continued trend from the pre-pandemic pattern (the counterfactual).

Figure 2. Rates of mental health care use split by all mental health care, general physician, and psychiatrist visits, for anxiety disorders.

a. All mental health care visits

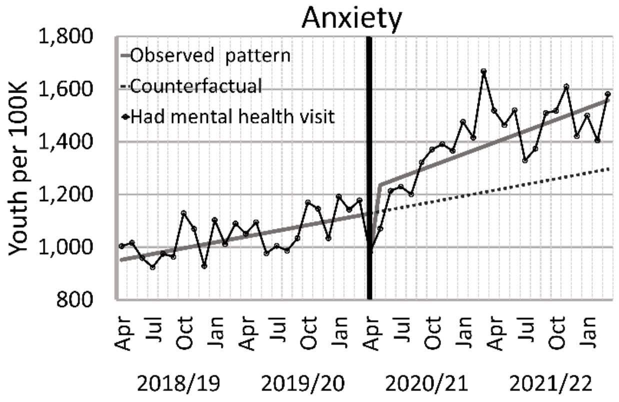

b. General physician visits

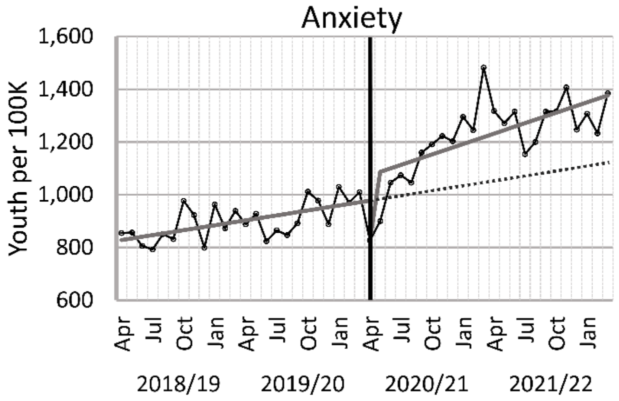

c. Psychiatrist visits

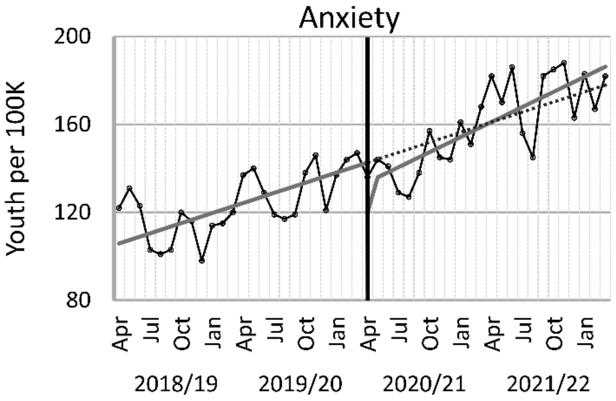

The black line with points is the actual rate. Points are monthly, but labels only shown quarterly. Solid grey lines are regression lines for the observed patterns. The vertical black line marks the start of the COVID-19 pandemic. The dashed line is the continued trend from the pre-pandemic pattern (the counterfactual).

Figure 3. **Rates of mental health care use split by all mental health care, general physician, and psychiatrist visits, for ADHD.**

a. All mental health care visits

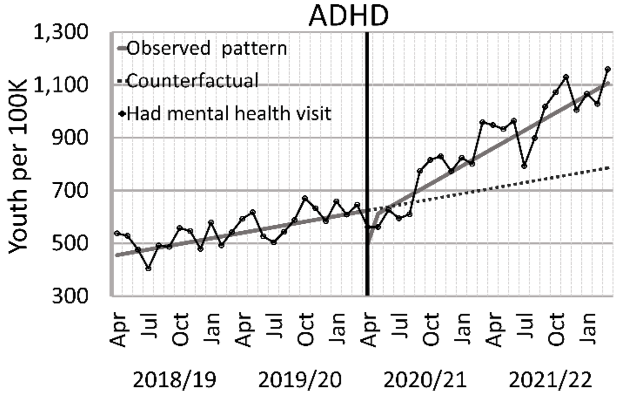

b. General physician visits

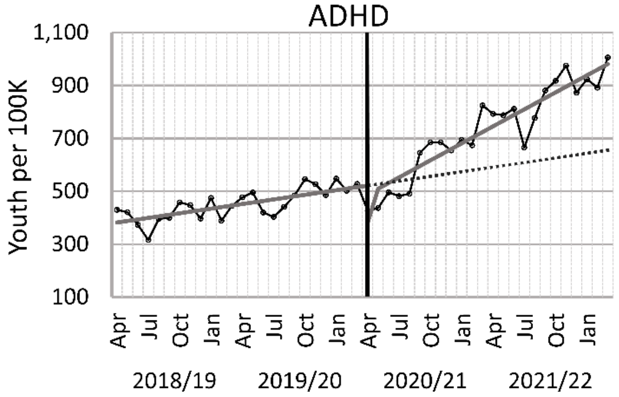

c. Psychiatrist visits

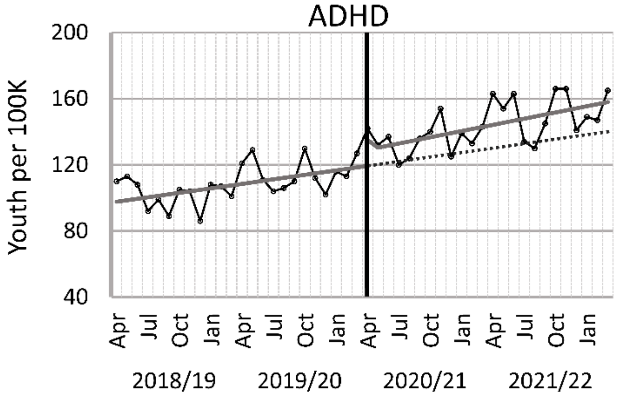

The black line with points is the actual rate. Points are monthly, but labels only shown quarterly. Solid grey lines are regression lines for the observed patterns. The vertical black line marks the start of the COVID-19 pandemic. The dashed line is the continued trend from the pre-pandemic pattern (the counterfactual).

Figure 4. **Rates of mental health care use split by all mental health care, general physician, and psychiatrist visits, for substance use disorders.**

a. All mental health care visits

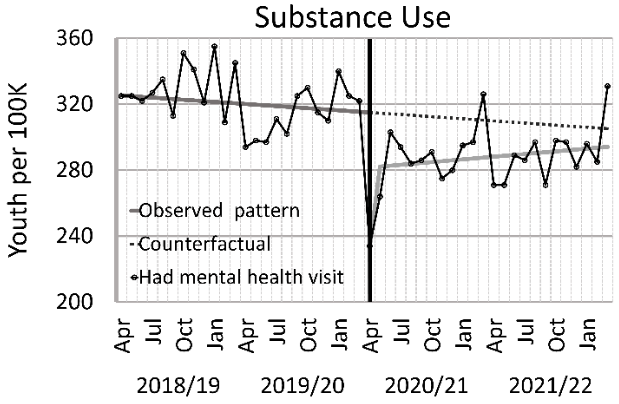

b. General physician visits

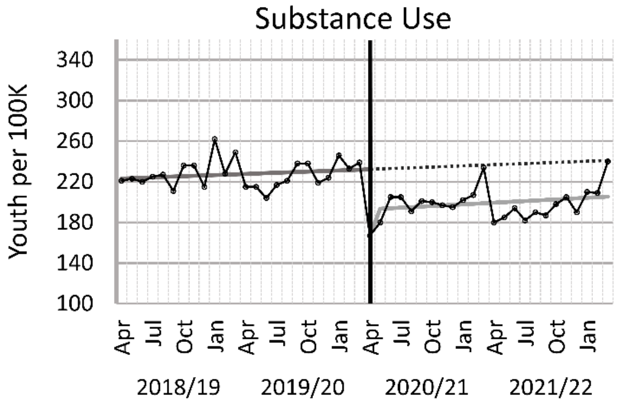

c. Psychiatrist visits

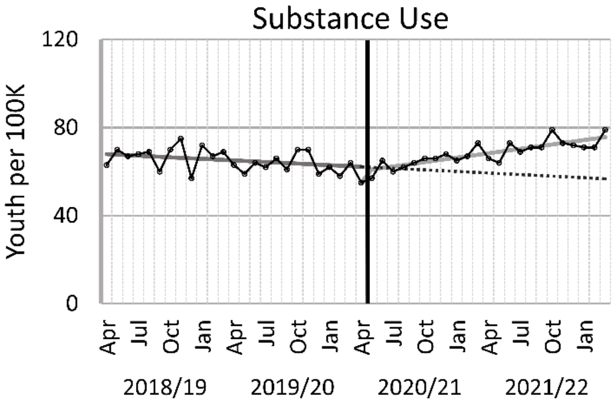

The black line with points is the actual rate. Points are monthly, but labels only shown quarterly. Solid grey lines are regression lines for the observed patterns. The vertical black line marks the start of the COVID-19 pandemic. The dashed line is the continued trend from the pre-pandemic pattern (the counterfactual).

Figure 5. **Rates of mental health care use split by all mental health care, general physician, and psychiatrist visits, for adjustment disorders.**

a. All mental health care visits

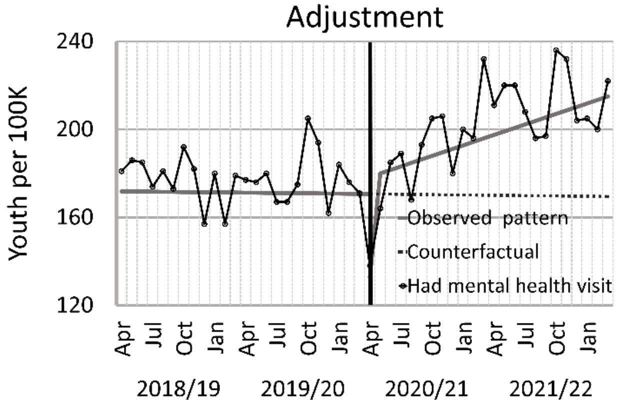

b. General physician visits

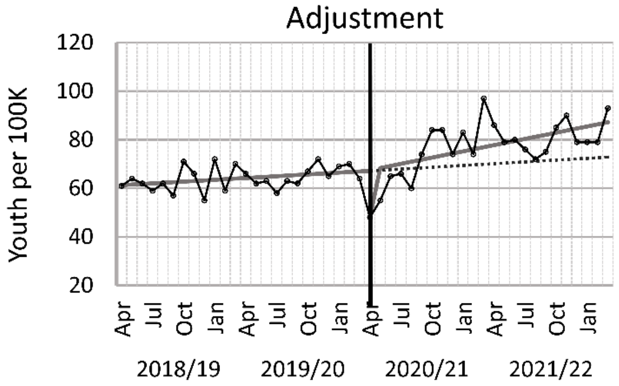

c. Psychiatrist visits

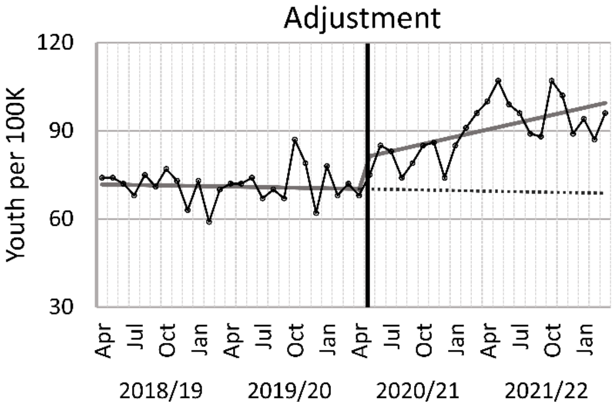

The black line with points is the actual rate. Points are monthly, but labels only shown quarterly. Solid grey lines are regression lines for the observed patterns. The vertical black line marks the start of the COVID-19 pandemic. The dashed line is the continued trend from the pre-pandemic pattern (the counterfactual).

Figure 6. **Rates of mental health care use split by all mental health care, general physician, and psychiatrist visits, for schizophrenia-related disorders.**

a. All mental health care visits

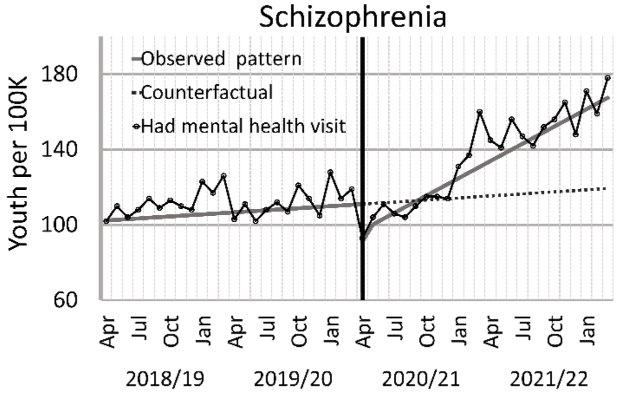

b. General physician visits

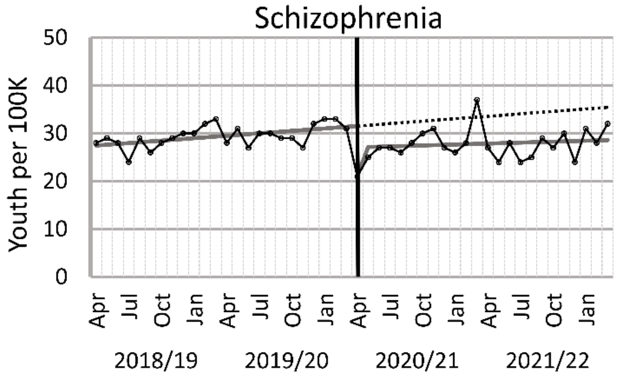

c. Psychiatrist visits

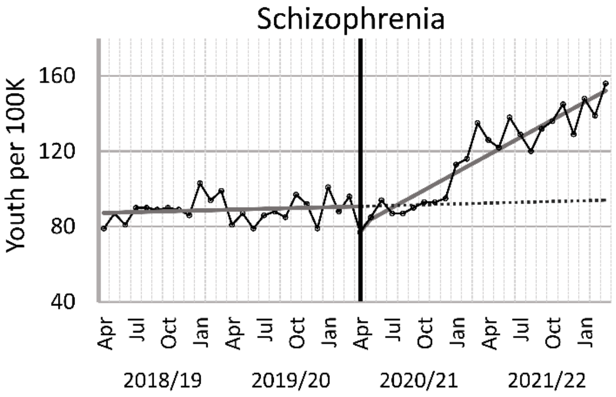

The black line with points is the actual rate. Points are monthly, but labels only shown quarterly. Solid grey lines are regression lines for the observed patterns. The vertical black line marks the start of the COVID-19 pandemic. The dashed line is the continued trend from the pre-pandemic pattern (the counterfactual).

Figure 7. **Rates of mental health care use split by all mental health care, emergency room, and hospitalization visits, for self-harm.**

a. All mental health care visits

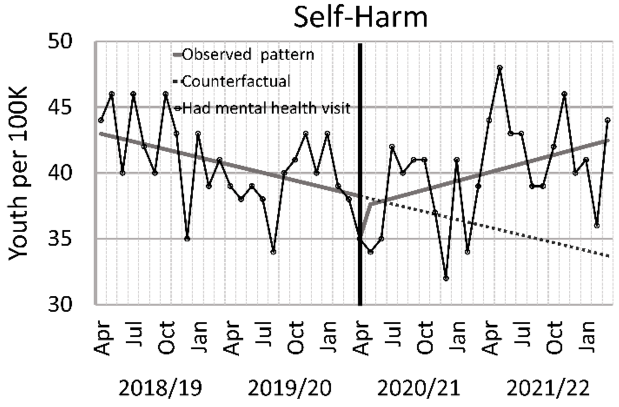

b. Emergency Room visits

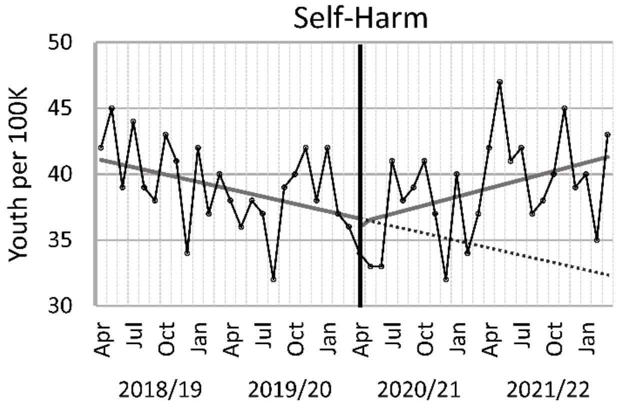

c. Hospitalizations

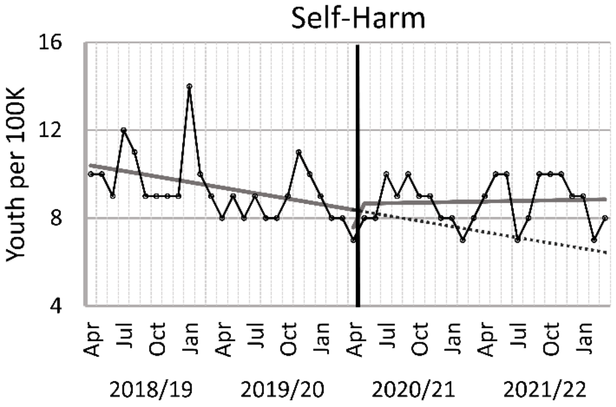

The black line with points is the actual rate. Points are monthly, but labels only shown quarterly. Solid grey lines are regression lines for the observed patterns. The vertical black line marks the start of the COVID-19 pandemic. The dashed line is the continued trend from the pre-pandemic pattern (the counterfactual).
